# Effects of parathyroid hormone rhPTH(1–84) on phosphate homeostasis and vitamin D metabolism in hypoparathyroidism: REPLACE phase 3 study

**DOI:** 10.1007/s12020-016-1141-0

**Published:** 2016-10-12

**Authors:** Bart L. Clarke, Tamara J. Vokes, John P. Bilezikian, Dolores M. Shoback, Hjalmar Lagast, Michael Mannstadt

**Affiliations:** 1 Division of Endocrinology, Diabetes, Metabolism, and Nutrition, Mayo Clinic, E18-A, 200 1st Street SW, Rochester, MN 55905 USA; 2 Section of Endocrinology, University of Chicago Medicine, 5841 South Maryland Avenue, MC1027, Chicago, IL 60637 USA; 3 Division of Endocrinology, College of Physicians and Surgeons, Columbia University, 630 W 168th Street, Room 864, New York, NY 10032 USA; 4 Endocrine Research Unit, San Francisco Department of Veterans Affairs Medical Center, University of California, 1700 Owens Street, San Francisco, CA 94158 USA; 5 NPS Pharmaceuticals, Inc., 300 Shire Way, Lexington, MA 02421 USA; 6 Endocrine Unit, Massachusetts General Hospital and Harvard Medical School, 50 Blossom Street, Thier-1123, Boston, MA 02114 USA

**Keywords:** Hypoparathyroidism, Phosphate, rhPTH(1–84), Parathyroid hormone, Vitamin D

## Abstract

In hypoparathyroidism, inappropriately low levels of parathyroid hormone lead to unbalanced mineral homeostasis. The objective of this study was to determine the effect of recombinant human parathyroid hormone, rhPTH(1–84), on phosphate and vitamin D metabolite levels in patients with hypoparathyroidism. Following pretreatment optimization of calcium and vitamin D doses, 124 patients in a phase III, 24-week, randomized, double-blind, placebo-controlled study of adults with hypoparathyroidism received subcutaneous injections of placebo or rhPTH(1–84) (50 µg/day, titrated to 75 and then 100 µg/day, to permit reductions in oral calcium and active vitamin D doses while maintaining serum calcium within 2.0–2.2 mmol/L). Predefined endpoints related to phosphate homeostasis and vitamin D metabolism were analyzed. Serum phosphate levels decreased rapidly from the upper normal range and remained lower with rhPTH(1–84) (*P* < 0.001 vs. placebo). At week 24, serum calcium–phosphate product was lower with rhPTH(1–84) vs. placebo (*P* < 0.001). rhPTH(1–84) treatment resulted in significant reductions in oral calcium dose compared with placebo (*P* < 0.001) while maintaining serum calcium. After pretreatment optimization, baseline serum 25-hydroxyvitamin D (25[OH]D) and 1,25-dihydroxyvitamin D (1,25[OH]_2_D) levels were within the normal range in both groups. After 24 weeks, 1,25(OH)_2_D levels were unchanged in both treatment groups, despite significantly greater reductions in active vitamin D dose in the rhPTH(1–84) group. In hypoparathyroidism, rhPTH(1–84) reduces serum phosphate levels, improves calcium–phosphate product, and maintains 1,25(OH)_2_D and serum calcium in the normal range while allowing significant reductions in active vitamin D and oral calcium doses.

## Introduction

Hypoparathyroidism is a rare disorder of unbalanced mineral homeostasis resulting from absent or inappropriately low levels of endogenous parathyroid hormone (PTH) [1–3]. Although hypocalcemia is a classic hallmark of hypoparathyroidism, hyperphosphatemia, and hypomagnesemia may also occur [4]. PTH acts directly on bone, increasing turnover, and resulting in the release of calcium and phosphate into the circulation [4, 5]. In the kidney, PTH stimulates renal reabsorption of calcium, promotes phosphate excretion in the urine, and enhances the conversion of 25-hydroxyvitamin D (25[OH]D) to the active 1,25-dihydroxyvitamin D (1,25[OH]_2_D) metabolite, which increases transepithelial transport of dietary calcium and phosphate from the intestine [4–6]. In concert, these effects help maintain normal serum calcium and phosphate levels.

With standard conventional management, the goal is to maintain serum calcium at the low end of the normal range while attempting to avoid both hypocalcemia and hypercalciuria as well as hyperphosphatemia, which can increase the calcium–phosphate product [4, 5]. Despite the clinical feasibility of therapy with oral calcium and active vitamin D (calcitriol or alfacalcidol), albeit used often in large amounts, important complications can ensue. Conventional therapy can lead to hypercalciuria, which in turn can result in nephrolithiasis and nephrocalcinosis, and irreversible kidney damage [4, 5, 7]. Active vitamin D metabolites do not replace the phosphaturic action of PTH and might exacerbate hyperphosphatemia by enhancing intestinal phosphate absorption [4]. Elevated serum phosphate levels may increase the serum calcium–phosphate product, thereby raising the risk for precipitation of calcium–phosphate. In patients with advanced renal disease, elevated serum phosphate levels and higher serum calcium–phosphate product were associated with coronary calcifications and increased mortality risk [8, 9]. In patients with hypoparathyroidism, calcification complexes have been reported in soft tissues, particularly the brain, lens, and kidney, but also the vasculature and other tissues [5, 7, 10–12]. Recent guidelines recommend maintaining calcium–phosphate product levels below 4.4 mmol^2^/L^2^ (55 mg^2^/dL^2^) to reduce the risk of ectopic calcifications and advise consideration of a low phosphate diet and, in certain cases, phosphate binders as needed to control phosphate levels [12–14].

In patients with hypoparathyroidism, daily subcutaneous (SC) treatment with recombinant human PTH (rhPTH(1–84), PTH rDNA; NATPARA^®^; Shire-NPS Pharmaceuticals, Inc., Lexington, MA, USA) has the potential to reproduce some of the physiologic effects of PTH compared with conventional therapy, such as lowering serum phosphate levels, promoting conversion of 25(OH)D to active 1,25(OH)_2_D, and improving renal tubular reabsorption of calcium. rhPTH(1–84) is identical to native human PTH [15, 16]. Treatment of hypoparathyroidism with rhPTH(1–84) has been studied in several recently published clinical trials [16–21]. In short, these studies demonstrate that treatment with rhPTH(1–84) is associated with reduced requirements for oral calcium and active vitamin D while maintaining control of symptoms, and some studies provide evidence for improved quality of life. The pivotal registration study for rhPTH(1–84), REPLACE, was a 24-week, randomized, double-blind, placebo-controlled, phase III, multicenter study that demonstrated that once-daily SC injections of rhPTH(1–84) reduced both oral calcium and active vitamin D requirements by ≥50 % from baseline levels while maintaining albumin-corrected total serum calcium levels in the target range (1.9 mmol/L to laboratory upper limit of normal) at Week 24 [19]. 53 % of patients who received rhPTH(1–84) achieved this endpoint compared with 2 % who received placebo (*P* < 0.0001). Complete independence from calcitriol or alfacalcidol and reduction of daily calcium doses to ≤500 mg/day while maintaining serum calcium in the target range was achieved by 43 % of the rhPTH(1–84) group compared with 5 % of the placebo group (*P* < 0.0001).

In this report, we present additional relevant results from the largest randomized, placebo-controlled trial completed in this endocrine-deficiency disease population. This information provides a greater understanding of the effects of rhPTH(1–84) beyond the previously reported composite primary endpoint of the REPLACE study [19]. Using predefined individual REPLACE clinical study exploratory endpoints, relevant serum and urine data collected during the trial were analyzed for the effects of rhPTH(1–84) on phosphate homeostasis and vitamin D metabolism.

## Materials and methods

### Study design

Detailed inclusion and exclusion criteria have been reported by Mannstadt et al. [19]. Briefly, patients between 18 and 85-years-old were defined as having hypoparathyroidism based on hypocalcemia and documented PTH levels below the lower limit of the normal range at least twice within the previous 12 months [19]. The study was conducted in 32 outpatient centers in accordance with Good Clinical Practice guidelines and the Declaration of Helsinki, and all study patients provided informed consent. This study is registered with ClinicalTrials.gov **(**NCT00732615) and EU clinical trials register (EudraCT 2008-005063-34).

During an optimization period over the first 2–16 weeks of the study, active vitamin D (calcitriol or alfacalcidol) and calcium doses were adjusted, as previously described [19]. During the optimization phase, native vitamin D doses were adjusted as follows: patients with serum 25(OH)D levels below the lower limit of normal (<75 nmol/L) were supplemented with vitamin D_3_ at 2000 IU/day until levels reached the normal range (75 nmol/L‒250 nmol/L), patients with serum 25(OH)D levels in the normal range received maintenance vitamin D_3_ at 400 IU/day, and patients with serum 25(OH)D levels above the normal range did not receive vitamin D_3_.

After calcium and active vitamin D doses were stable for a 2-week period, which established the baseline doses, patients were randomized in a 1:2 ratio to receive once-daily SC injections of placebo or 50 μg rhPTH(1–84). The 24-week treatment period began with a stepwise titration phase that allowed for 2 uptitrations of rhPTH(1–84) dose, if needed, to reduce active vitamin D first and then oral calcium, as previously described [19]. The titration phase was followed by a maintenance phase for the remainder of the treatment period; rhPTH(1–84) could be downtitrated at any time, if necessary, but not uptitrated. Adjustments were permitted in active vitamin D and oral calcium doses to maintain serum calcium in the target range and to improve hypercalciuria at any time during the treatment period. During the treatment phase, patients received at least 400 IU/day of vitamin D_3_; patients with serum 25(OH)D levels below the lower limit of normal received additional vitamin D_3_ as necessary to bring levels into the normal range. rhPTH(1–84) was discontinued at the conclusion of 24 weeks, whereby baseline oral calcium and active vitamin D were resumed, and patients were followed up for 4 weeks.

### Assessments

The assessments for serum phosphate levels were scheduled at baseline and Weeks 1, 2, 3, 4, 5, 6, 8, 12, 16, 20, and 24, and 24-hour urine phosphate measurements were done at baseline and Weeks 3, 5, 6, 8, 12, 16, and 24. Serum phosphate was measured 24 h after the last injection of rhPTH(1–84) or placebo, as part of a 24-analyte serum chemistry panel that required fasting for ≥8 h before testing. Reported serum phosphate values in mg/dL were converted to International System of Units (mmol/L) using a standard conversion factor of 0.323 [22]. Serum levels of 25(OH)D were assessed at baseline and 24 h after the last injection of rhPTH(1–84) or placebo at Weeks 2, 4, 6, 12, and 24 by chemiluminescent assay. Serum levels of 1,25(OH)_2_D were also assessed at baseline and 24 h after the last study drug injection at Weeks 12 and 24 by extraction chromatography followed by radioreceptor assay.

### Statistical analysis

Treatment group differences for serum and urine phosphate were compared using an analysis of covariance model, with actual change as the dependent variable, treatment as a factor, and baseline value as a covariate. Treatment group differences for active vitamin D dose were compared using an analysis of covariance model, with percentage change as the dependent variable, treatment as a factor, and the baseline dose as a covariate. A converted active vitamin D value was used, in which 2 doses of alfacalcidol were equated to 1 dose of calcitriol [23]. The albumin-corrected serum calcium levels were measured and calculated as previously described [19]. The *P* values for inter-treatment group comparison of differences were calculated from the least squares mean difference and standard error.

During the US regulatory review of the rhPTH(1–84) application for the treatment of patients with hypoparathyroidism, the sponsor was requested to re-evaluate the REPLACE data based on 124 study patients from 31 centers in North America and Europe, after data from one clinical site was removed. Mannstadt et al. had reported that the 134 patients with hypoparathyroidism randomized in the REPLACE study met the primary efficacy endpoint and had a generally safe profile [19]. After all comparisons and analyses were recalculated, there was no change in the primary endpoint efficacy and safety conclusions, and the US Prescribing Information cites the data analysis from 124 study patients [24]. This exploratory analysis report also evaluated the matching 124-patient data set, and for completeness in data analysis provides the re-analyzed primary calcium values in the supplementary material.

## Results

Of the 124 patients analyzed, 84 were in the rhPTH(1–84) group and 40 were in the placebo group, reflecting the 2:1 randomization. Table 1 summarizes key patient demographics and baseline characteristics. 79 % of patients were women. The mean ± SD age was 47.3 ± 12.7 years, body mass index was 29.2 ± 6.1 kg/m^2^, and duration of hypoparathyroidism was 13.6 ± 10.3 years. Twelve patients discontinued treatment before study completion: 5 in the rhPTH(1–84) group and 7 in the placebo group. Of these, two patients, both in the rhPTH(1–84) group, withdrew because of an adverse event (one patient had several adverse events, some of which were judged to be treatment related; the other patient had a cerebrovascular accident that was not thought to be related to treatment).

**Table 1:** Patient population characteristics at baseline (after optimization of active vitamin D and calcium doses)

Variable	rhPTH(1–84) (*n* = 84)	Placebo (*n* = 40)	Reference range (If applicable)
Mean (range) age, years	46.6 (19–74)	48.9 (21–73)	
Women (men), *n*	65 (19)	33 (7)	
Mean ± SD body mass index, kg/m^2^	29.3 ± 6.4	28.9 ± 5.3	
Duration (range) of hypoparathyroidism, years	14.6 (2–50)	11.6 (2–38)	
Baseline characteristics after optimization period			
Prescribed calcium, *n* (%)			
0–2000 mg/day	57 (68)	29 (72.5)	
>2000 mg/day	27 (32)	11 (27.5)	
	Mean ± SD	Mean ± SD	
Prescribed native vitamin D,^a^ IU/day	2217 ± 6875	1467 ± 3971	
Prescribed calcitriol,^b^ µg/day	0.9 ± 0.5	0.8 ± 0.4	
Serum 25(OH)D levels, nmol/L	105.5 ± 37.0	110.9 ± 47.4	75–250
Serum 1,25(OH)_2_D levels, pmol/L	87.6 ± 55.1	85.0 ± 29.9	39–156
Calcium parameters			
Serum calcium,^c^ mmol/L	2.1 ± 0.2	2.2 ± 0.2	2.1–2.6
Urinary calcium, mmol/24 h	9.0 ± 4.8	8.4 ± 4.3	1.3–7.5
Phosphate parameters			
Serum phosphate, mmol/L	1.5 ± 0.2	1.5 ± 0.2	0.8–1.6
Urinary phosphate, mmol/24 h	34.2 ± 14.2	33.7 ± 12.0	12.9–42.0
Serum magnesium, mmol/L	0.83 ± 0.08	0.84 ± 0.07	0.65–1.05
Calcium–phosphate product, mmol^2^/L^2^	3.22 ± 0.54	3.29 ± 0.52	<4.4

*rhPTH* recombinant human parathyroid hormone, *ULN* upper limit of normal

^a^ Among those patients receiving native vitamin D (rhPTH(1–84), *n* = 61; placebo, *n* = 24)

^b^ Alphacalcidol dose is converted to calcitriol dose based on a conversion factor of 2 alphacalcidol equals 1 calcitrio

^c^ Albumin-corrected total serum calcium

### Phosphate levels

**Fig. 1:**
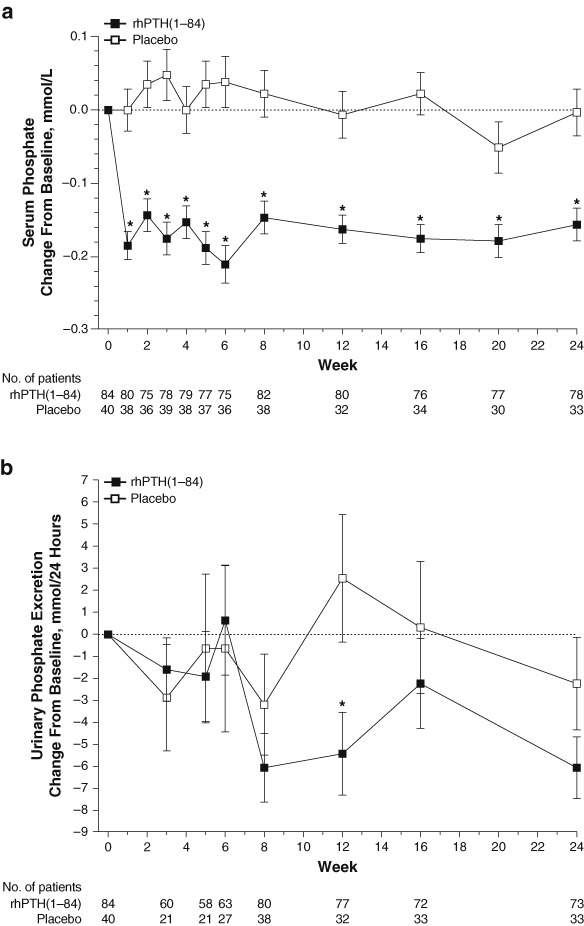
Change from baseline in serum phosphate **a** and urinary phosphate **b** during treatment with rhPTH(1–84) or placebo in patients with hypoparathyroidism. Change from baseline values is least squares mean ± SE. rhPTH = recombinant human parathyroid hormone. *P ≤ 0.004 **a** or *P* = 0.03 **b** for the mean change from baseline for the rhPTH(1–84) vs. placebo

At screening, mean ± SD serum phosphate level for the study group was in the upper normal range at 1.5 ± 0.3 mmol/L (normal range, 0.8–1.6 mmol/L [25]). After optimization, baseline serum phosphate levels were similar for both treatment groups (1.5 ± 0.2 mmol/L for each). The time course data in Fig. 1a demonstrate that the serum phosphate levels in patients receiving rhPTH(1–84), measured 24 h after the last injection, decreased rapidly from baseline. At Week 1 (the first on-treatment study visit), serum phosphate had declined by 0.2 ± 0.02 mmol/L (least squares mean ± SE). In contrast, in the placebo group, serum phosphate was unchanged at Week 1 (change from baseline: 0.0 ± 0.03 mmol/L; *P* < 0.001 vs. rhPTH(1–84)). This initial decrease in serum phosphate with rhPTH(1–84) was maintained between Weeks 1 and 24, and was significantly lower compared with the placebo arm in which levels remained near baseline. Furthermore, at every study visit between Weeks 1 and 24, serum phosphate levels remained stable and were significantly lower in the rhPTH(1–84) treatment arm compared with the placebo arm. At Week 24, serum phosphate declined by 0.2 ± 0.02 mmol/L in rhPTH(1–84)-treated patients and did not change in placebo-treated patients (change from baseline: 0.0 + 0.03 mmol/L; *P* < 0.001).

Baseline 24-h urine phosphate excretion levels were similar in both treatment groups (rhPTH (1–84), 34.2 ± 14.2 mmol/24 h; placebo, 33.7 ± 12.0 mmol/24 h). In contrast to serum phosphate measurements, urinary phosphate data showed substantial variability, and no significant change between the groups was apparent until Week 12 (Fig. 1b **)**. At Week 12, the significant decrease in urinary phosphate levels in the rhPTH(1–84) group was 5.4 ± 1.9 mmol/24 h compared with an increase of 2.4 ± 3.0 mmol/24 h in the placebo group (least squares mean ± SE; *P* = 0.03). At Week 24, 24-h urinary phosphate excretion levels had decreased in both treatment arms relative to baseline; the decrease was 6.1 ± 1.4 mmol/24 h in patients receiving rhPTH(1–84) compared with a decrease of 2.4 ± 2.1 mmol/24 h in patients receiving placebo (*P* = 0.15).

### Calcium–phosphate product levels

**Fig. 2:**
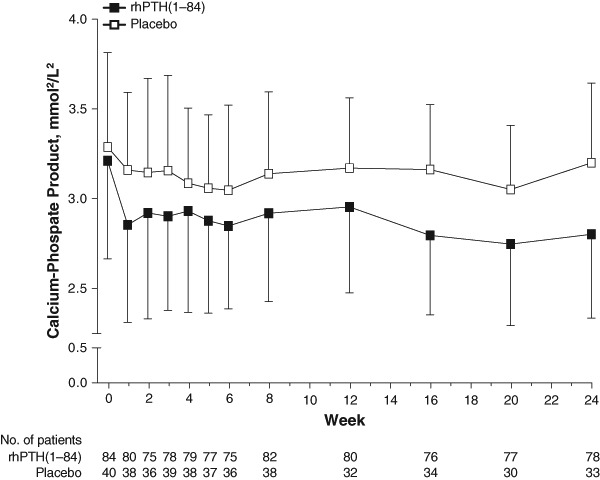
Time course of change in calcium–phosphate product during treatment with rhPTH(1–84) or placebo in patients with hypoparathyroidism measured before the next injection. Values are mean ± SD. rhPTH = recombinant human parathyroid hormone

Prescribed calcium dose, albumin-corrected serum calcium and urinary calcium at baseline and Week 24, and time course data for serum calcium are presented in Supplementary Tables 1 and 2; these data confirm the observed reductions reported in the primary endpoint study analysis [19]. At baseline after optimization, mean ± SD calcium–phosphate product levels were similar for both treatment groups (rhPTH(1–84), 3.2 ± 0.5 mmol^2^/L^2^; placebo, 3.3 ± 0.5 mmol^2^/L^2^; Fig. 2). Calcium–phosphate product levels decreased rapidly in the rhPTH(1–84) treatment arm; at Week 1, calcium–phosphate product levels declined to 2.9 ± 0.5 mmol^2^/L^2^. In contrast, the levels in the placebo group remained steady; Week 1 levels were 3.2 ± 0.4 mmol^2^/L^2^. These values were generally maintained over the remainder of the study: at Week 24, calcium–phosphate product levels were 2.8 ± 0.5 mmol^2^/L^2^ in patients receiving rhPTH(1–84) and 3.2 ± 0.4 mmol^2^/L^2^ in patients receiving placebo. The least squares mean ± SE change in the calcium–phosphate product from baseline to Week 24 between the treatment arms was significantly different: there was a decrease of 0.4 ± 0.1 mmol^2^/L^2^ in the rhPTH(1–84) group and a decrease of only 0.1 ± 0.1 mmol^2^/L^2^ in the placebo group (*P* < 0.001).

### Native vitamin D

**Fig. 3:**
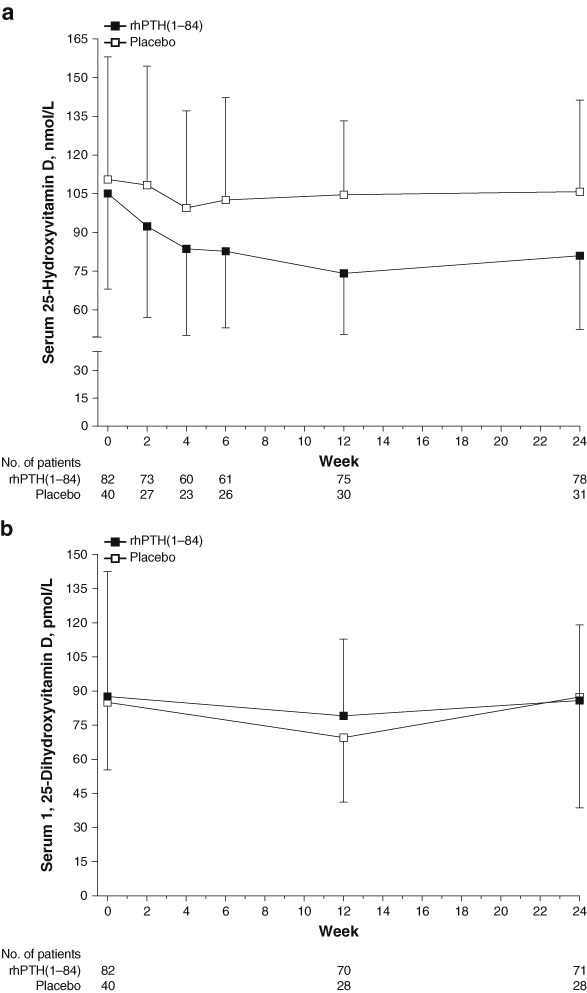
Time course of change in serum 25-hydroxyvitamin D **a** and 1,25-dihydroxyvitamin D **b** during treatment with rhPTH(1–84) or placebo in patients with hypoparathyroidism. Values are mean ± SD. rhPTH = recombinant human parathyroid hormone

When the mean daily dose is calculated from the total dose over the treatment period, patients on rhPTH(1–84) were on a higher dose of oral native vitamin D compared with patients in the placebo group (1487 ± 2127 IU/day vs. 917 ± 1312 IU/day; mean ± SD). After optimization, baseline serum levels of 25(OH)D were within the normal range and were similar for both treatment groups (Table 1 and Fig. 3a). Among patients receiving rhPTH(1–84), serum 25(OH)D levels declined steadily between baseline and Week 12, and then stabilized, whereas in patients receiving placebo, serum 25(OH)D levels decreased only slightly within the first 4 weeks and stayed near baseline levels. At Weeks 2 and 12, mean ± SD serum 25(OH)D levels declined by 14.1 ± 18.6 nmol/L and 32.3 ± 31.8 nmol/L, respectively, in the rhPTH(1–84) group and remained below baseline until Week 24 (change from baseline: −24.9 ± 39.2 nmol/L). In contrast, there was no change in the placebo group on serum 25(OH)D levels (mean ± SD change from baseline at Week 24: −3.1 ± 33.6 nmol/L).

### Activated vitamin D

After optimization, baseline serum levels of 1,25(OH)_2_D were within the normal range and were similar for both treatment groups (Table 1 and Fig. 3b). The mean ± SD baseline active vitamin D dose was 0.9 ± 0.5 µg/day in patients receiving rhPTH(1–84) and 0.8 ± 0.4 µg/day in patients receiving placebo; 67 and 62 % of patients treated with rhPTH(1–84) or placebo, respectively, were taking >0.5 μg/day calcitriol or >1.0 μg/day alfacalcidol. At the beginning of the treatment period, active vitamin D doses were reduced per study protocol; in the initial 2 weeks, the reductions were 51 ± 4 % and 51 ± 6 % (least squares mean ± SE percentage change from baseline values), for rhPTH(1–84)-treated and placebo-treated patients, respectively (Supplementary Fig. 1). Patients in the rhPTH(1–84) group continued dose reductions until Week 8 (90 ± 4 % reduction) and ended treatment at Week 24 with a 77 ± 4 % reduction from baseline. In the placebo group, the dose decreased by 63 ± 5 % between baseline and Week 6 and ended treatment at Week 24 with a 35 ± 7 % reduction from baseline. By Week 24, dose reductions from baseline were significantly greater for patients receiving rhPTH(1–84) compared with patients receiving placebo (*P* < 0.001); 87 % of patients receiving rhPTH(1–84) had a ≥50 % reduction in active vitamin D dose compared with 45 % of patients receiving placebo. There were fewer measurements of serum levels of 1,25(OH)_2_D compared with serum 25(OH)D levels. Serum levels of 1,25(OH)_2_D remained stable throughout the study without major changes in both groups (change from baseline at Week 24 with rhPTH(1–84) and placebo: increase of 2.6 ± 52.3 pmol/L and decrease of 0.8 ± 45.0 pmol/L, respectively).

Among patients who achieved independence from oral active vitamin D (62 %, rhPTH(1–84); 24 %, placebo), serum 1,25(OH)_2_D was maintained with rhPTH(1–84) treatment but decreased with placebo (change from baseline at Week 24, increase of 3.2 ± 52.4 pmol/L, and decrease of 25.0 ± 27.9 pmol/L, respectively, for these analytes).

## Discussion

This analysis of exploratory endpoints from the pivotal phase III REPLACE study provides further evidence that rhPTH(1–84) therapy for patients with hypoparathyroidism is efficacious and has different effects from conventional treatment on serum phosphate, calcium–phosphate product, and vitamin D metabolites [19]. Here, we provided detailed time course data on the rapid changes in phosphate, calcium–phosphate product, and vitamin D metabolite levels with rhPTH(1–84) treatment.

On conventional therapy, serum phosphate levels often exceed the normal range during the day in patients with hypoparathyroidism [21]. Indeed, mean serum phosphate levels were in the upper range of normal in both groups at baseline, reflecting the lack of the phosphaturic effect of PTH [5]. Elevated serum phosphate levels are associated with a greater risk of soft tissue calcifications, likely via increases in calcium–phosphate product levels [5, 7, 10–12]. In addition, preclinical studies suggest that high levels of serum phosphate may directly promote vascular calcification via osteochondrogenic conversion of vascular smooth muscle cells [26]. Among patients with end-stage renal disease or cardiovascular disease, elevated serum phosphate levels correlate with increased mortality risk [9, 27]. Our results show that, compared with placebo, rhPTH(1–84) treatment significantly decreased serum phosphate levels. It is noteworthy that the reductions reported here in serum phosphate levels and calcium–phosphate product were noticeable despite the fact that the measurements were made 24 h after the last PTH injection, a time point that reflects a minimal reduction in serum phosphate and calcium–phosphate product [21]. In a phase I pharmacodynamic (PD) and pharmacokinetic (PK) study conducted in patients with hypoparathyroidism, Clarke et al. reported that reductions in serum phosphate and calcium–phosphate product occur rapidly following rhPTH(1–84) injection [21] and were maximal at 12 h following injection with 50–100 µg rhPTH(1–84).

The decline in serum phosphate levels in the rhPTH(1–84) group was not accompanied by a corresponding increase in urinary phosphate excretion. Transient changes in urinary phosphate excretion may have been missed in this study because of the timing of assessments, and we may have captured only the new steady state. In the phase I PK/PD study, serum phosphate decreased to a nadir at 5 h after injection of rhPTH(1–84), which was associated with a corresponding increase in urinary fractional excretion of phosphate over the same time course [21]. Similarly, Sikjaer et al. showed that urinary phosphate excretion increased over the first 8 h post-injection of rhPTH(1–84), followed by a return to near-baseline levels over the next 16 h [28]. In the present study, the first post-baseline assessment of urinary phosphate was conducted after 1 week of rhPTH(1–84) treatment had been completed and 24 h after the last study drug dose. By 1 week of treatment with rhPTH(1–84), new steady-state serum phosphate levels may already be established, such that no further increases in urinary phosphate would be required to maintain the lower serum phosphate concentrations. Furthermore, while the study protocol did not restrict patients’ dietary intake or the administration of phosphate binders, no patient took phosphate binders during the study. Thus, fluctuations in dietary phosphate during the study may also have contributed to the variability in urinary phosphate. Finally, compared with other time points, fewer patients had urine phosphate levels assessed at the early study visits (up to Week 6). The smaller sample sizes at these early time points may have contributed to the lack of consistent between-group differences at these time points. Nonetheless, the urinary phosphate data presented here might inform future studies investigating phosphate dynamics in hypoparathyroidism.

Although no prospective data exist in the hypoparathyroidism population, a somewhat arbitrary threshold of 4.4 mmol^2^/L^2^ (55 mg²/dL²) is generally accepted as the upper limit of normal, and recent treatment guidelines include maintenance of calcium–phosphate product below that level as a goal of hypoparathyroidism management [13, 14]. Overall, our analysis showed that levels of calcium–phosphate product decreased to a greater extent in the rhPTH(1–84) group than in the placebo group. Furthermore, calcium–phosphate product levels declined rapidly with rhPTH(1–84) within 1 week of treatment. Decreases in calcium–phosphate product were evident with rhPTH(1–84) treatment despite the fact that serum calcium levels remained stable during the study. The observed reductions in calcium–phosphate product were likely driven primarily by rhPTH(1–84)-mediated decreases in serum phosphate. Together, these data suggest that, unlike conventional management strategies with oral calcium and active vitamin D therapy, treatment with rhPTH(1–84) improves phosphate levels and calcium–phosphate product in patients with hypoparathyroidism. Thus, rhPTH(1–84) has the potential to reduce complications, such as soft tissue calcification, compared with conventional therapy by more effectively restoring the full range of physiologic activities elicited by the endogenous hormone.

The 2–16-week optimization period before randomization in the REPLACE study ensured that patients had optimized their conventional treatment with oral calcium and calcitriol or alfacalcidol and had normal serum 25(OH)D and magnesium levels. As a result, serum levels for both 25(OH)D and 1,25(OH)_2_D were within the normal range at baseline. Despite significant reductions in oral active vitamin D or even complete independence of active vitamin D in a subgroup, patients treated with rhPTH(1–84) maintained serum levels of the active vitamin D metabolite 1,25(OH)_2_D in the normal range. In contrast, placebo-treated patients who eliminated oral active vitamin D experienced decreases in serum 1,25(OH)_2_D. This reflects the physiologic role of PTH to enhance conversion of 25(OH)D to 1,25(OH)_2_D. In addition, other metabolic pathways may be contributing to the turnover of 25(OH)D in patients treated with rhPTH(1–84), including catabolism by cytochrome P450 (CYP) 24A1 to generate 24,25(OH)_2_D [29]. In some cell types, CYP24A1 is known to be activated by PTH [29]. Because 25(OH)D constitutes the substrate for conversion to active vitamin D, care has to be taken to keep 25(OH)D levels within the normal range in these patients. Consequently, over the course of the study, patients receiving rhPTH(1–84) had higher daily requirements for supplemental native vitamin D than patients receiving placebo, although the variability was large. Thus, administration of rhPTH(1–84) may permit reduction or elimination of oral calcium and active vitamin D supplementation, but native vitamin D supplementation should be continued. In practice, careful monitoring of serum 25(OH)D levels, as well as adjustments of oral native vitamin D supplementation as necessary, is warranted for patients receiving rhPTH(1–84).

The results reported here are consistent with other published data using different study designs and dosing regimens of rhPTH(1–84) [18, 20]. Cusano et al. reported that, among 27 patients treated with rhPTH(1–84) 100 μg every other day, serum phosphate levels decreased from 1.4 ± 0.6 mmol/L at baseline to approximately 1.3 mmol/L at 4 years and remained in the normal range throughout the study [18]. In a study of add-on rhPTH(1–84) therapy to standard treatment, Sikjaer et al. reported that serum phosphate levels decreased from 1.1 mmol/L at baseline to less than 1.0 mmol/L in 32 patients receiving rhPTH(1–84) 100 μg every day at 24 weeks [20]. Overall, median serum 25(OH)D was 72 nmol/L at baseline and did not change during the study. Serum 1,25(OH)_2_D was 98 pmol/L at baseline and increased rapidly in patients receiving rhPTH(1–84). Similar to our observations, levels in the Sikjaer study remained stable throughout that study, despite the marked dose reduction in active vitamin D (median reduction, 50 and 0 % in patients treated with rhPTH(1–84) and placebo, respectively; *P* < 0.001).

As previously mentioned, a limitation in the REPLACE study was that serum phosphate and 1,25(OH)_2_D levels were measured 24 h after the last injection; therefore, we likely missed the maximal decrease of serum phosphate and calcium–phosphate product and the maximal increase in 1,25(OH)_2_D levels during the first 8 h.

The REPLACE study demonstrated that daily SC treatment with rhPTH(1–84) allowed reductions in the large amounts of oral calcium and active vitamin D generally required for symptom management without increasing urinary calcium excretion [19]. This current analysis extends the primary findings of the REPLACE study and provides further mechanistic evidence that therapy with rhPTH(1–84), in contrast to conventional therapy with oral calcium and active vitamin D, mimics the action of endogenous PTH in patients with hypoparathyroidism [12]. In summary, treatment with rhPTH(1–84) addresses the multiple physiologic mineral and vitamin D abnormalities that occur in hypoparathyroidism by decreasing serum phosphate levels toward the mid-normal range, improving the calcium–phosphate product, and maintaining serum 1,25(OH)_2_D levels in the normal range. This latter benefit will be maintained if careful monitoring of serum 25(OH)D levels and adjustments in oral native vitamin D supplementation as necessary are performed by the treating endocrinologist.

## Electronic supplementary material

Supplementary Information

Supplementary Information
