# Supplementary material for: Effects of parathyroid hormone rhPTH(1–84) on phosphate homeostasis and vitamin D metabolism in hypoparathyroidism: REPLACE phase 3 study
Source: Endocrine. 2016 Oct 12;55(1):273–82. doi: 10.1007/s12020-016-1141-0 (PMC5225224; doi:10.1007/s12020-016-1141-0)
Supplement: Supplementary file 1 — Supplementary Information [file 12020_2016_1141_MOESM1_ESM.docx]

**Supplemental Material:**

**Title: Effects of Parathyroid Hormone rhPTH(1-84) on Phosphate** **Homeostasis and Vitamin D Metabolism in Hypoparathyroidism: REPLACE Phase 3 Study**

**Journal:** *Endocrine*

**Authors Information**

Bart L. Clarke,^1^ Tamara J. Vokes,^2^ John P. Bilezikian,^3^ Dolores M. Shoback,^4^ Hjalmar Lagast,^5*^ Michael Mannstadt^6^

^1^Division of Endocrinology, Diabetes, Metabolism, and Nutrition, Mayo Clinic, E18-A, 200 1st Street SW, Rochester, MN 55905, USA; ^2^Section of Endocrinology, University of Chicago Medicine, 5841 South Maryland Avenue, MC1027, Chicago, IL 60637, USA; ^3^Division of Endocrinology, College of Physicians and Surgeons, Columbia University, 630 W 168th Street, Room 864, New York, NY 10032, USA; ^4^Endocrine Research Unit, San Francisco Department of Veterans Affairs Medical Center, University of California, 1700 Owens Street, San Francisco, CA 94158, USA; ^5^NPS Pharmaceuticals, Inc., 300 Shire Way, Lexington, MA 02421, USA; ^6^Endocrine Unit, Massachusetts General Hospital and Harvard Medical School, 50 Blossom Street, Thier-1123, Boston, MA 02114, USA

*****Current affiliation is OnPoint Clinical Consulting, Bridgewater, NJ 08807, USA

**Correspondence**: Bart L. Clarke, MD; Division of Endocrinology, Diabetes, Metabolism, and Nutrition Mayo Clinic, E18-A; 200 1st Street SW, Rochester, MN 55905, USA, email: clarke.bart@mayo.edu; phone: 507-266-4322; fax: 507-284-5745

**Supplemental Table 1. Calcium Parameters at Baseline and Week 24**

|  | **rhPTH(1-84)**  **(n=84)^c^** | | | | **Placebo**  **(n=40)^g^** | | |
| --- | --- | --- | --- | --- | --- | --- | --- |
|  | **Baseline,**  **Mean ± SD** | **Week 24,**  **Mean ± SD** | **Change From Baseline at Week 24,**  **LS Mean ± SE** | **Baseline,**  **Mean ± SD** | | **Week 24,**  **Mean ± SD** | **Change From Baseline at Week 24,**  **LS Mean ± SE** |
| Prescribed calcium dose, mg/day | 2171±1434 | 987±1000 | –1126±104^d^ | 1978±926 | | 1932±977 | –113±161 |
| Serum calcium,^a^ mmol/L | 2.13±0.21 | 2.09±0.23 | –0.04±0.03^e^ | 2.15±0.16 | | 2.09±0.23 | –0.05±0.04 |
| Urinary calcium,^b^ mmol/24 hours | 9.0±4.8 | 6.9±4.4 | −1.9±0.4^f^ | 8.5±4.3 | | 6.1±3.5 | −2.5±0.7 |

LS=least squares; rhPTH=recombinant human parathyroid hormone.

^a^Albumin-corrected total serum calcium; at screening, the mean ± SD albumin-corrected total serum calcium level for the study group as a whole was 2.0±0.2 mmol/L.

^b^At screening, the mean ± SD urine calcium excretion for the study group as a whole was 6.7±4.4 mmol/24 hours.

^c^Except n=79 at Week 24 for calcium dose, n=78 at Week 24 for serum calcium, and n=74 at Week 24 for urinary calcium.

^d^*P*<0.001 vs placebo.

^e^*P*=0.84 vs placebo.

^f^*P*=0.44 vs placebo.

^g^Except n=33 at Week 24.

**Supplemental Table 2. Albumin-corrected Total Serum Calcium Values Over Time**

| **Week** | **rhPTH(1-84)**  **(n=84)** | **Placebo**  **(n=40)** |
| --- | --- | --- |
| 0 | 2.13±0.21 | 2.15±0.16 |
| 1 | 2.16±0.20 | 2.07±0.14 |
| 2 | 2.17±0.15 | 2.04±0.14 |
| 3 | 2.20±0.18 | 2.03±0.13 |
| 4 | 2.21±0.22 | 2.04±0.11 |
| 5 | 2.22±0.19 | 1.99±0.15 |
| 6 | 2.23±0.22 | 1.95±0.16 |
| 8 | 2.18±0.23 | 2.05±0.14 |
| 12 | 2.20±0.21 | 2.07±0.12 |
| 16 | 2.13±0.21 | 2.06±0.16 |
| 20 | 2.10±0.23 | 2.07±0.09 |
| 24 | 2.09±0.24 | 2.09±0.23 |

Unit values are mmol/L (mean ± SD).

^a^Except n=80 at Week 1; n=75 at Weeks 2 and 6; n=79 at Week 3 and 4; n=78 at Weeks 5 and 24; n=82 at Week 8; n=81 at Week 12; and n=77 at Weeks 16 and 20.

^b^Except n=38 at Weeks 1, 4 and 8; n=36 at Weeks 2 and 6; n=39 at Week 3; n=37 at Week 5; n=32 at Week 12; n=34 at Week 16; n=30 at Week 20 and n=33 at Week 24.

**Supplemental Figure Legend**

**Supplemental Fig. 1** Time course of change in active vitamin D dose during treatment with rhPTH(1-84) or placebo in patients with hypoparathyroidism. Percentage change from baseline values is least squares mean ± SE. rhPTH=recombinant human parathyroid hormone. **P*≤0.01 for the mean percentage change from baseline for rhPTH(1-84) vs placebo
