# Supplementary figures and images for: Effects of parathyroid hormone rhPTH(1–84) on phosphate homeostasis and vitamin D metabolism in hypoparathyroidism: REPLACE phase 3 study
Source: Endocrine. 2016 Oct 12;55(1):273–82. doi: 10.1007/s12020-016-1141-0 (PMC5225224; doi:10.1007/s12020-016-1141-0)

■ rhPTH(1-84)  
□ Placebo

Daily Dose of Active Vitamin D  
Change From Baseline, %

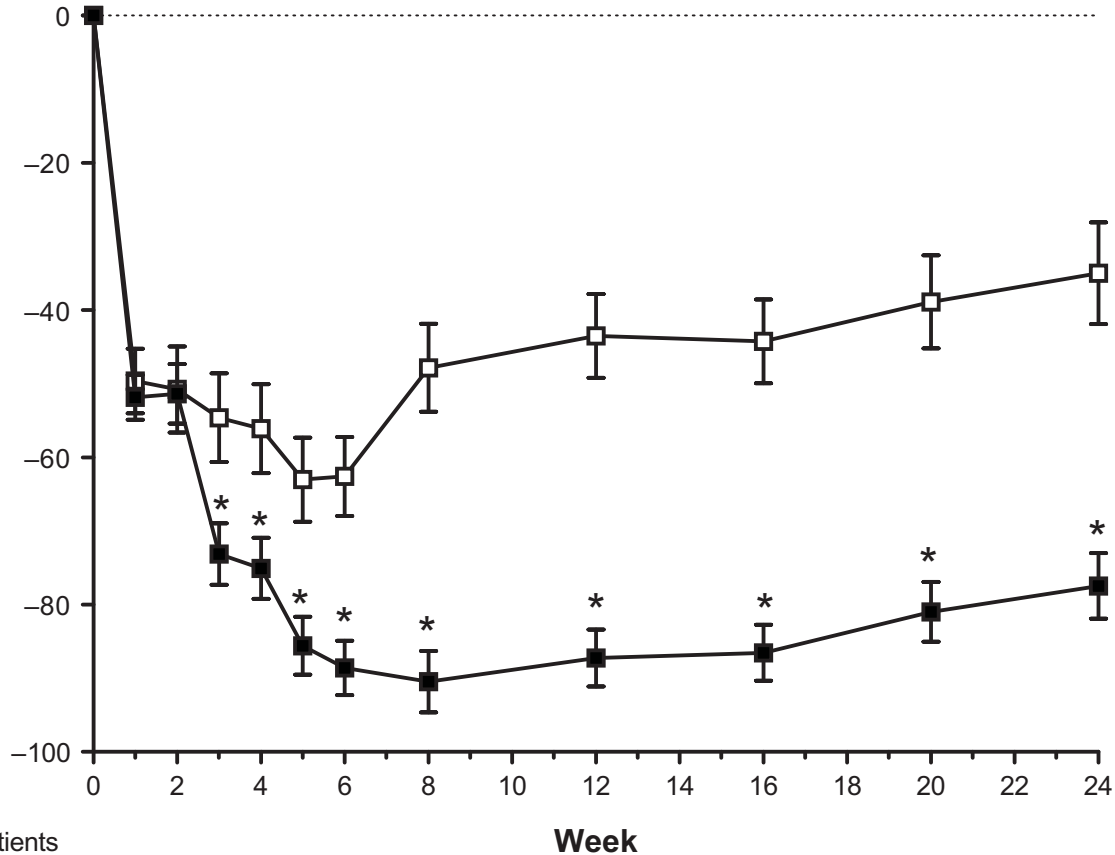

No. of patients

|             |    |    |    |    |    |    |    |    |    |    |    |    |
|-------------|----|----|----|----|----|----|----|----|----|----|----|----|
| rhPTH(1-84) | 84 | 84 | 84 | 84 | 84 | 84 | 84 | 82 | 82 | 81 | 79 | 79 |
| Placebo     | 40 | 40 | 40 | 40 | 40 | 40 | 40 | 40 | 38 | 36 | 33 | 33 |

Supplement: Supplementary file 2 — Supplementary Information [file 12020_2016_1141_MOESM2_ESM.pdf]
